# Incidence of food insecurity among adults and older adults diagnosed with coronavirus disease 2019: a longitudinal study, Rio Grande, 2021-2023

**DOI:** 10.1590/S2237-96222026v35e20250935.en

**Published:** 2026-08-02

**Authors:** Thais dos Anjos Velho, Lilia Schug de Moraes, Bianca Languer Vargas, Mirelle de Oliveira Saes, Suele Manjourany Silva Duro, Romina Buffarini

**Affiliations:** 1 Universidade Federal do Rio Grande, de Pós-Graduação em Ciências da Saúde, Rio Grande, RS, Brazil Universidade Federal do Rio Grande de Pós-Graduação em Ciências da Saúde Rio Grande RS Brazil; 2 Universidade Federal de Pelotas, Programa de Pós-Graduação em Enfermagem, Pelotas, RS, Brazil Universidade Federal de Pelotas Programa de Pós-Graduação em Enfermagem Pelotas RS Brazil

**Keywords:** Food Insecurity, COVID-19, Adult, Aged, Longitudinal Studies, Inseguridad Alimentaria, COVID-19, Adulto, Anciano, Estudios Longitudinales

## Abstract

**Objective:**

To assess the incidence of food insecurity over a one-and-a-half-year period in Rio Grande, Rio Grande do Sul, and to investigate risk factors, considering adults and older adults who had coronavirus disease 2019.

**Methods:**

This is a longitudinal study, with data collected at two time points: 2021 and 2022-2023. Food insecurity was assessed using the reduced version of the Brazilian Food Insecurity Scale (Escala Brasileira de Insegurança Alimentar, EBIA). The following were considered as exposure variables: sex, age, race/color, marital status, educational level, family income, income situation, and employment status during the pandemic. The variables were described using absolute and relative frequencies, and proportions were compared using the Chi-square test. Associated factors were estimated using Poisson regression with robust variance adjustment.

**Results:**

Among the 1,231 individuals analyzed, the incidence of food insecurity during the period was 20.1%. In the adjusted analysis, female sex (incidence ratio [IR] 1.53; 95% confidence interval [95%CI] 1.21; 1.95), lower educational levels (IR 1.75; 95%CI 1.26; 2.41 and IR 1.54; 95%CI 1.16; 2.04), lower family income (IR 2.01; 95%CI 1.17; 3.47 and IR 2.53; 95%CI 1.54; 4.15), and marked income reduction during the pandemic (IR 1.51; 95%CI 1.15; 1.98) were risk factors associated with the incidence of food insecurity.

**Conclusion:**

The results showed that 2 out of every 10 individuals developed food insecurity over the follow-up period, which spanned the pandemic and the subsequent period, with greater risk among individuals in more socially vulnerable groups.

## Resumen

**Objetivo:**

Evaluar la incidencia de la inseguridad alimentaria durante un período de un año y medio en Rio Grande, Rio Grande do Sul, e investigar factores de riesgo, considerando a adultos y personas mayores que presentaron la enfermedad por coronavirus de 2019.

**Métodos:**

Se trata de un estudio longitudinal, con recolección de datos en dos momentos: 2021 y 2022-2023. La inseguridad alimentaria se evaluó mediante la versión reducida de la Escala Brasileña de Inseguridad Alimentaria (Escala Brasileira de Insegurança Alimentar, EBIA). Se consideraron variables de exposición: sexo, edad, raza/color de piel, estado civil, nivel de escolaridad, ingreso familiar, situación de ingreso y situación laboral durante la pandemia. Las variables fueron descritas mediante frecuencias absolutas y relativas, y las proporciones se compararon mediante la prueba de Chi-cuadrado. Los factores asociados se estimaron mediante regresión de Poisson con ajuste de varianza robusta.

**Resultados:**

Entre los 1.231 individuos analizados, la incidencia de inseguridad alimentaria en el período analizado fue del 20,1%. En el análisis ajustado, se observó que el sexo femenino (razón de incidencia [RI] 1,53; intervalo de confianza del 95% [IC95%] 1,21; 1,95), menores niveles de escolaridad (RI 1,75; IC95% 1,26; 2,41 y RI 1,54; IC95% 1,16; 2,04), menores niveles de ingreso familiar (RI 2,01; IC95% 1,17; 3,47 y RI 2,53; IC95% 1,54; 4,15) y reducción marcada del ingreso durante la pandemia (RI 1,51; IC95% 1,15; 1,98) fueron factores de riesgo asociados a la incidencia de inseguridad alimentaria.

**Conclusión:**

Los resultados mostraron que dos de cada diez individuos desarrollaron inseguridad alimentaria a lo largo del período de seguimiento, que abarcó la pandemia y el período posterior, con mayor riesgo entre individuos pertenecientes a grupos socialmente más vulnerables.

**Ethical aspects**

This research respected ethical principles, having obtained the following approval data:

Research ethics committee: Universidade Federal do Rio Grande

Opinion number: 4,375,697

Approval date: 3/11/2020

Certificate of submission for ethical appraisal: 39081120.0.0000.5324

Informed consent record: Obtained from all participants before data collection.

## Introduction

At the end of 2019, a new respiratory virus was identified in China, severe acute respiratory syndrome coronavirus 2, the causative agent of coronavirus disease 2019^1^. Following the identification of the first cases and confirmation of efficient human-to-human transmission, the virus spread rapidly worldwide, and the World Health Organization declared a pandemic in March 2020. Brazil, in turn, adopted the recommendations and began following international protocols. This scenario triggered a series of crises (health, economic, political, and social), contributing to increased hunger worldwide^2^.

Food and nutritional insecurity occur when individuals lack continuous, permanent access to healthy, high-quality food in sufficient quantities, while respecting diversity, local culture, and sustainability. The Organic Law on Food and Nutritional Security (Law No. 11,346, dated 15 September 2006) establishes food and nutritional security as a basic right that should be guaranteed to all citizens, without compromising access to other essential needs^3^. Given that the pandemic reduced food production and distribution, it impacted both supply and demand, leading to a reduction in the population's purchasing power, especially among the most vulnerable households^4^.

In this context, the relationship between food insecurity and household income became evident, with low-income families facing the greatest difficulties in acquiring sufficient quantities of quality food daily^5^. The reduction in household income resulting from the pandemic also led to changes in the dietary habits of the adult population, such as increased consumption of ultra-processed foods and, in some cases, food insecurity due to extreme poverty, characterized by insufficient per capita income (below BRL 105.00)^6^.

In 2018, the Consumer Expenditure Survey (Pesquisa de Orçamentos Familiares, POF) showed that approximately 37% of Brazilian households were already experiencing food insecurity^7^. The survey used the Brazilian Food Insecurity Scale (Escala Brasileira de Insegurança Alimentar, EBIA) as a direct measure of perceived household food insecurity, categorized into three levels (mild, moderate, and severe). The study demonstrated that food insecurity affected the lives of millions of Brazilians, highlighting the importance of effective measures and policies, as well as social support, to reduce the impacts of hunger and ensure food and nutritional security for the population^7^.

According to data from the National Survey on Food Insecurity in the Context of the COVID-19 Pandemic in Brazil, conducted by the Brazilian Research Network on Food and Nutritional Sovereignty and Security (Rede Brasileira de Pesquisa em Soberania e Segurança Alimentar e Nutricional, Rede PENSSAN), 55% of Brazilian households experienced some degree of food insecurity, and 19 million people were affected by severe food insecurity (hunger) in late 2020^8^. In a study conducted in Rio Grande, Rio Grande do Sul, between August 2020 and February 2021, approximately one-third of households experienced food insecurity^9^.

This study sought to investigate further food insecurity in the context of the COVID-19 pandemic using two time points in a longitudinal design. In the Brazilian context, no studies were identified that assessed the incidence of food insecurity; the national literature has predominantly focused on prevalence estimates and specific populations^10^^-^^13^. The objectives of this study were therefore to assess the incidence of food insecurity over a one-and-a-half-year period, covering the pandemic and a subsequent period (between late 2022 and early 2023), in Rio Grande, and to analyze potential risk factors among adults and older adults diagnosed with coronavirus disease 2019.

## Methods

Study design

This longitudinal study used data from the SulCovid-19 study, a population-based survey that monitored the health of adults and older adults following coronavirus disease 2019 infection, conducted in Rio Grande, Rio Grande do Sul. 

Setting

Rio Grande is located in the extreme south of Brazil and had an estimated population of approximately 192,000 inhabitants in 2022, with a population density of 71.53 inhabitants/km² and a Human Development Index of 0.74.

Data from the SulCovid-19 study were obtained through structured interviews conducted with adults and older adults infected with coronavirus disease 2019 in Rio Grande. Data collection took place at two time points: (1) baseline study, from June to October 2021, with a sample of 2,920 individuals; (2) follow-up study, from October 2022 to May 2023, with a response rate of 66% (n=1,927). The average interval between the two waves of the study was approximately 1.5 years (17-18 months), based on the midpoints of the data collection periods.

Previously trained interviewers carried out data collection. Responses were collected electronically via the Research Electronic Data Capture system, tablets, and smartphone calls. The calls were recorded using a free mobile application (Callmaster) to ensure the safety of both the researcher and the respondent.

Participants

Sample identification was carried out using the list of confirmed coronavirus disease 2019 cases obtained from the municipality's epidemiological surveillance service. From this list, individuals who met the study eligibility criteria (n=3,822) were contacted by telephone. In addition to telephone interviews, interviewers could conduct data collection through household visits. In the study, only one individual was interviewed at home.

Eligible individuals were those aged ≥18 years, residing in Rio Grande, and diagnosed with coronavirus disease 2019 through reverse transcription polymerase chain reaction testing, between December 2020 and March 2021. Individuals with functional limitations and/or advanced neurological diseases that prevented them from responding to the questionnaire were excluded, as well as those in long-term care institutions or deprived of their liberty. Individuals who were not located after five attempts-one by telephone, one via WhatsApp, and three household visits-were considered losses.

Variables

The study outcome variable was the cumulative incidence of household food insecurity. For textual simplification, it will be referred to throughout the manuscript as "incidence". Exposure variables included sex, age, race/color, marital status, education, family income, change in income during the pandemic, and employment status.

Measurement

Household food insecurity was assessed using the reduced version of the EBIA, which consists of 5 questions. The instrument was validated to assess food insecurity in the Brazilian population and aims to identify perceptions of the presence/lack of food at the household level. In this study, food insecurity questions were administered to a single household member, the SulCovid-19 respondent. Food insecurity was considered present when the participant answered "yes" to at least one of the five items in the questionnaire.

The reduced five-question version was compared with the original 14-item EBIA (the gold standard) in a sample from Pelotas, Rio Grande do Sul, a neighboring state to Rio Grande^14^. The study showed high sensitivity (95.7%), 100% specificity, and excellent accuracy (97%), with results similar to those of the original version.

To calculate the cumulative incidence of food insecurity, the number of participants with food insecurity data in both the baseline and follow-up studies was first identified (n=1,899). Individuals who already presented the outcome at baseline (n=668) were excluded from the denominator, as they were not eligible to become new cases. The denominator consisted of individuals free of the outcome at the beginning of the period (n=1,231). Incident cases were then defined as those individuals who were not experiencing food insecurity at baseline but reported food insecurity at follow-up. Cumulative incidence was the proportion of these individuals who developed the outcome between baseline and follow-up.

Exposure variables were collected in the baseline study. For statistical analysis purposes, these variables were categorized as follows: sex (male, female), age group (18-34 years, 35-59 years, ≥60 years), race/color (White, Brown [Brazilian mixed-race], Black), marital status (married/with partner, single, separated, widowed), educational level (0-8 years of schooling, 9-11 years of schooling, ≥12 years of schooling), family income (less than BRL 1,000.00, BRL 1,001.00 to BRL 2,000.00, BRL 2,001.00 to BRL 4,000.00, above BRL 4,000.00), family income situation during the pandemic (stable, slight reduction, marked reduction), and employment status (employment maintained, pre-pandemic labor inactivity, and unemployment due to the pandemic).

The race/color variable was collected using the five categories of the Brazilian Institute of Geography and Statistics (Instituto Brasileiro de Geografia e Estatística, IBGE): White, Black, Asian, Brown, and Indigenous. Among individuals with data at baseline and follow-up, the percentage of individuals self-declared as Asian was 0.3%, and Indigenous 0.2%. No observations from these categories were eligible in the final analytical sample.

Sample size

The original sample comprised 2,920 individuals, of whom 1,927 were interviewed at follow-up. As the study used information from both stages, the analytical sample comprised 1,231 individuals with complete food insecurity data at baseline and follow-up who were at risk of developing food insecurity (Figure 1). 

Bias

Strategies to control bias were adopted before and during data collection. These strategies included careful selection of data-collection instruments, development of operational manuals, standardization of the interviewer team, conducting a pilot study, and rigorous control over data management and processing. To reduce losses to follow-up, the study adopted a two-stage active strategy. In telephone data collection, each eligible participant received up to five contact attempts, conducted on different days and at different times, followed by a standardized message via WhatsApp if no response was received. If the individual responded, the interview was scheduled by telephone or in person, as preferred. Participants who did not answer calls or respond to messages were referred to the next stage. In household data collection, a trained and properly equipped interviewer conducted at least one in-person visit to the households of those not interviewed in the telephone phase. Addresses that could not be located were classified as losses.

**Figure 1.**
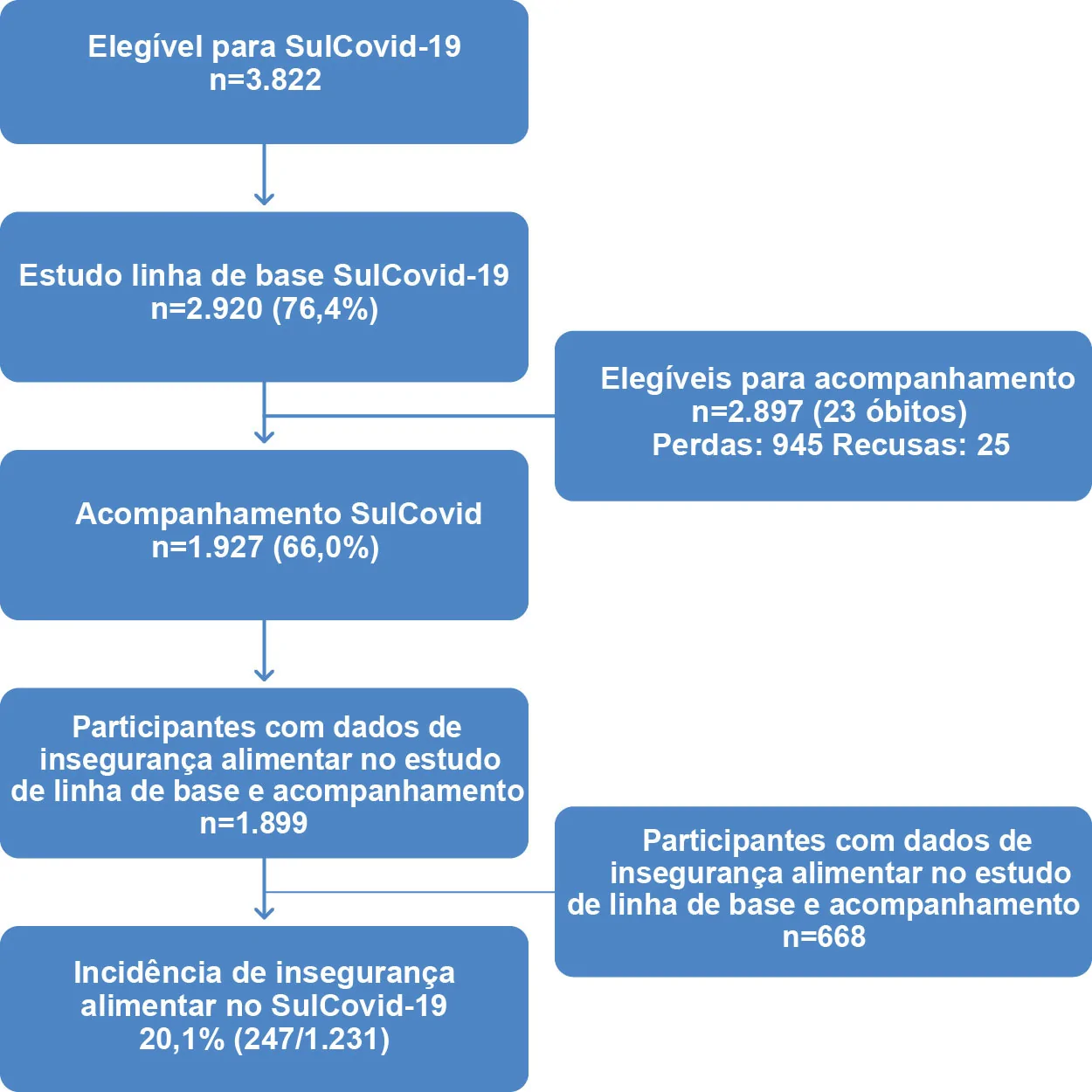
Flowchart of participant selection based on data from the SulCovid-19 study. Rio Grande, 2021-2023

At the time of analysis, to assess potential selection bias due to loss to follow-up, we compared participants who were followed up with those who were not among all individuals eligible in the baseline study (Supplementary table 1). The reference sample for this comparison comprised 2,897 participants, obtained after excluding 23 deaths from the baseline study (n=2,920). In the comparison between followed and non-followed participants, a higher proportion of women and individuals in older age groups (≥35 years) was observed among those followed up. At the same time, the other sociodemographic characteristics did not differ between the groups (Supplementary table 1).

Statistical methods

Variables were described using absolute and relative frequencies, and, for ordinal variables, proportions were compared using Pearson's Chi-square test or the Chi-square test for linear trend (Cochran-Armitage). The assessment of potential risk factors associated with the incidence of food insecurity was performed using crude and adjusted Poisson regression models with robust variance, estimating incidence ratios (IR) and their respective 95% confidence intervals (95%CI). To avoid adjustment for a potential mediator, independent variables were included in the adjusted model at four levels, according to a hierarchical model of determination^15^.

Independent variables were organized by their conceptual distance from the outcome and their potential for causal influence across levels. At the first level, demographic variables were included (sex, age, race/color); at the second level, marital status, educational level, and family income were included, representing socioeconomic determinants that the previous levels may partially explain; at the third level, the employment status variable was included; lastly, change in income during the pandemic was included, a factor that may have been conditioned by previous levels and may be directly related to recent conditions of access to food. Associations with p-value<0.050 were considered statistically significant. All analyses were performed using Stata 16.1 software (StataCorp).

## Results

Table 1 presents the sociodemographic characteristics of the analytical sample (n=1,231). Most participants were female (56.2%), nearly half were aged 35-59 years (52.3%), the majority self-identified as White (80.7%), and were married or living with a partner (64.2%). Regarding educational level, the largest proportion had 9-11 years of schooling (42.9%), and 57.3% reported a monthly family income of up to BRL 2,000. During the pandemic, nearly one-third (34.4%) reported some level of income reduction, while 4.1% became unemployed. Discrepancies in the total of variables in relation to the total number of observations in the analytical sample may be due to missing information for some variables: family income (10.7%), race/color (4.7%), employment status during the pandemic (3.6%), educational level (1.6%), income during the pandemic (2.4%), marital status (0.4%), and age (0.1%).

**Table 1:** Description of the analytical sample using data from the SulCovid-19 study. Rio Grande, 2021-2023 (n=1,231)

Variable	n (%)
Sex	
Male	539 (43.8)
Female	692 (56.2)
Age group (years)	
18-34	350 (28.5)
35-59	643 (52.3)
≥60	236 (19.2)
Race/color	
White	938 (80.7)
Brown (Brazilian mixed race)	132 (10.8)
Black	103 (8.5)
Marital status	
Married/living with a partner	787 (64.2)
Single	285 (23.2)
Separated	95 (7.8)
Widowed	59 (4.8)
Educational level (years of schooling)	
0-8	263 (21.8)
9-11	520 (42.9)
≥12	428 (35.3)
Family income (BRL)	
Up to 1,000	205 (18.7)
1,001-2,000	424 (38.6)
2,001-4,000	301 (27.4)
≥4,001	169 (15.3)
Income during pandemic	
Stability	792 (65.6)
Slight reduction	218 (18.1)
Marked reduction	196 (16.3)
Employment status during pandemic	
Employment maintained	79 (65.7)
Pre-pandemic labor inactivity	358 (30.2)
Unemployment due to the pandemic	49 (4.1)

**Table 2:** Incidence of food insecurity in a sample of adults and older adults according to sociodemographic variables. Sul Covid-19, Rio Grande, 2022-2023 (n=1,231)

Variable	Food insecurity (%)	p-value
Overall	20.1	
Sex		<0.001
Male	15.6	
Female	23.6	
Age group (years)		0.789
18-34	18.9	
35-59	20.7	
≥60	19.9	
Race/color		0.258
White	19.5	
Brown (Brazilian mixed race)	18.9	
Black	26.2	
Marital status		0.194
Married/living with a partner	18.2	
Single	22.8	
Separated	25.3	
Widowed	20.3	
Educational level (years of schooling)		0.003
0-8	23.6	
9-11	21.9	
≥12	15.0	
Family income (BRL)		<0.001
Up to 1,000	22.4	
1,001-2,000	26.2	
2,001-4,000	16.3	
≥4,001	9.5	
Income during pandemic		0.001
Stability	17.6	
Slight reduction	22.9	
Marked reduction	27.0	
Employment status during pandemic		0.283
Employment maintained	19.1	
Pre-pandemic labor inactivity	22.1	
Unemployment due to the pandemic	26.5	

The incidence of food insecurity in the total sample was 20.1% (Table 2). When analyzed by sex, it was significantly higher among women (23.6%) than among men (p-value<0.001), and among individuals with 0-8 years of schooling (23.6%) than among those with higher educational levels (15.0%) (p-value 0.006). No significant differences were observed according to age group (p-value 0.789), race/color (p-value 0.258), or marital status (p-value 0.194). A higher incidence of food insecurity was observed among individuals with a monthly family income of up to BRL 2,000 (p-value<0.001). Incidence showed a progressive decrease with increasing education levels (p-value for linear trend 0.003). A gradient was observed for income change during the pandemic, with a higher incidence of food insecurity among those who reported a marked reduction, followed by a slight reduction, compared to those who maintained stable income (p-value for linear trend 0.001). It was also found that the incidence of food insecurity was higher among participants who became unemployed as a result of the pandemic (26.5%), followed by those who were labor inactive (22.1%); however, this association was not statistically significant (p-value 0.283) (Table 2).

In both crude and adjusted analyses, the variables sex, educational level, family income, and change in income during the pandemic were associated with the incidence of food insecurity (Table 3). The results showed that women (RR 1.53; 95%CI 1.21; 1.95), those with fewer years of schooling (0-8 years: RR 1.75; 95%CI 1.26; 2.41; 9-11 years: RR 1.54; 95%CI 1.16; 2.04), those with lower family income (up to R$ 1,000: RR 2.01; 95%CI 1.17; 3.47; between BRL 1,001-2,000: RR 2.53; 95%CI 1.54; 4.15), and those who reported a marked reduction in income during the pandemic (RR 1.51; 95%CI 1.15; 1.98) had a higher risk of food insecurity (Table 3).

## Discussion 

 Data from the present study revealed that among adults and older adults diagnosed with coronavirus disease 2019 in Rio Grande, two out of every ten developed food insecurity over a period of approximately one and a half years (between 2021 and late 2022/early 2023), even after the most critical period of the COVID-19 pandemic. Women, those with lower educational levels, those with lower family income, and those who reported a reduction in income during the pandemic had a higher risk of incident food insecurity. The scarcity of longitudinal studies assessing the incidence of food insecurity during the pandemic limited direct comparisons with this study's analyses. In a study conducted in the southern region of the state, based on four epidemiological surveys of coronavirus disease 2019, approximately one-third of households experienced food insecurity in 2020^16^. Although these were cross-sectional data, this evidence corroborated the findings of the present study and contextualized the magnitude of the problem in the region.

The National Survey conducted by the Brazilian Research Network on Food and Nutritional Sovereignty and Security (Inquérito Nacional da Rede Brasileira de Pesquisa em Soberania e Segurança Alimentar e Nutricional) showed that more than half of Brazilian households (55.2%) experienced some degree of food insecurity in the pandemic context, but without incidence data^17^. Across all regions of the state, income inequality was the primary factor contributing to food and nutritional security conditions, and lower-income families were the most vulnerable to food insecurity, as evidenced in the present study^8^. In a study conducted in Porto Alegre, 33% of the individuals interviewed were experiencing food and nutritional insecurity, and the most affected groups included female heads of household, individuals with low educational levels, and families with incomes below three minimum wages^18^.

The literature has shown that the social and economic impacts of the pandemic contributed to an increase in household food insecurity^19^. This context also highlighted Brazil's historical structural inequalities^20^^,^^21^, reflecting structural racism, patriarchal social organization, and the weakening of public policies that limit access to employment, income, and education opportunities. The reduction in income, increased unemployment, and the absence of assistance policies and programs intensified the pre-existing social inequality and food and nutritional insecurity^7^^,^^17^. The dismantling of public policies on food and nutritional security in Brazil^22^ contributed to the worsening of this situation. The extinction of the National Council on Food and Nutritional Security (Conselho Nacional de Segurança Alimentar e Nutricional, CONSEA), the dismantling of the Interministerial Chamber on Food and Nutritional Security (Câmara Interministerial de Segurança Alimentar e Nutricional, CAISAN), and the reduction of programs in this area compromised the funding and implementation of essential actions, weakening food sovereignty and increasing social vulnerability^4^.

**Table 3:** Unadjusted and adjusted relative risk (RR) and 95% confidence intervals (95%CI) for the incidence of food insecurity according to sociodemographic variables in the study. SulCovid-19, Rio Grande, 2021-2023 (n=1,899)

Variable	Unadjusted		Adjusted	
	RR (95%CI)	p-value	RR (95%CI)	p-value
Sex		<0.001		<0.001
Male	Ref.			
Female	1.51 (1.19; 1.92)		1.53 (1.21; 1.95)	
Age group (years)		0.79		0.761
18-34	0.95 (0.68; 1.32)		0.92 (0.66; 1.31)	
35-59	1.03 (0.77; 1.39)		1.02 (0.76; 1.39)	
≥60	Ref.			
Race/color		0.237		0.192
White	Ref.		Ref.	
Brown (Brazilian mixed race)	0.97 (0.67; 1.41)		0.99 (0.67; 1.45)	
Black	1.34 (0.95; 1.90)		1.38 (0.97; 1.95)	
Marital status		0.187		0.107
Married	Ref.		Ref.	
Single	1.26 (0.97; 1.62)		1.30 (1.00; 1.69)	
Separated	1.39 (0.95; 2.02)		1.34 (0.91; 1.94)	
Widowed	1.11 (0.66; 1.89)		0.84 (0.47; 1.49)	
Educational level (years of schooling)		0.007		0.001
0-8	1.58 (1.15; 2.16)		1.75 (1.26; 2.41)	
9-11	1.47 (1.11; 1.94)		1.54 (1.16; 2.04)	
≥12	Ref.		Ref.	
Family income (BRL)		<0.001		<0.001
Up to 1,000	2.37 (1.39; 4.03)		2.01 (1.17; 3.47)	
1,001-2,000	2.77 (1.69; 4.53)		2.53 (1.54; 4.15)	
2,001-4,000	1.72 (1.01; 2.92)		1.66 (0.97; 2.83)	
≥4,001	Ref.		Ref.	
Income during the pandemic		0.005		0.009
Stability	Ref.		Ref.	
Slight reduction	1.31 (0.98; 1.74)		1.28 (0.95; 1.99)	
Marked reduction	1.54 (1.17; 2.02)		1.51 (1.15; 1.98)	
Employment status during pandemic		0.271		0.715
Employment maintained	Ref.		Ref.	
Pre-pandemic labor inactivity	1.15 (0.91; 1.47)		0.90 (0.66; 1.21)	
Unemployment due to the pandemic	1.39 (0.85; 2.25)		1.05 (0.62; 1.81)	

The findings of this study have practical implications, particularly in the context of the COVID-19 pandemic, whose social impacts, although already known and debated, continue to have repercussions across society and contribute to the social rights agenda. These results provide important support for local and national strategies to improve public policies on food and nutritional security, health, and social assistance, especially for the most vulnerable groups. Research has indicated that economic recovery needs to be accompanied by intersectoral measures that ensure universal and equitable access to food and the guarantee of social rights^4^^,^^23^.

The study had limitations, particularly related to losses to follow-up. The higher proportion of women and older individuals among those followed up may have influenced the estimates, as these groups are more vulnerable to food insecurity^5^^,^^24^. The fact that the sample comprised only individuals infected with coronavirus disease 2019 also limits the external validity of these findings to populations not affected by COVID-19. A similar limitation is that the study was conducted in Rio Grande, which limits the external validity of the results to other regions with different socioeconomic and cultural contexts. The results should therefore be interpreted with caution. Underreporting of COVID-19 cases in Brazil^25^ may have contributed to identifying fewer infected individuals in the original study sample. This may limit the representativeness of the sample in relation to the total number of infected individuals in the population.

It should be noted, however, that this does not compromise the estimates of food insecurity among the included participants, since the study was based exclusively on cases confirmed by RT-PCR and widely captured by municipal surveillance. The rigorous contact procedures, including multiple telephone calls, WhatsApp messages, and household visits, are believed to have substantially minimized underreporting in the study. Finally, it is important to emphasize that, in addition to the variables analyzed in this study, other factors such as social support networks^26^^,^^27^, receipt of emergency aid^28^^,^^29^, and household arrangements^24^ have been associated with food insecurity and should be explored in future studies. These aspects may directly affect Brazilian families' ability to cope with situations of vulnerability.

Among the strengths, the longitudinal design stands out, as it allowed us to demonstrate the incidence of food insecurity over time and the possibility of documenting regional food insecurity, despite the sample limitation, conducted during the pandemic and the early post-pandemic period, enabling an assessment of the situation in Rio Grande during an important moment of health crisis.

The data from this study showed the incidence of food insecurity among adults and older adults diagnosed with coronavirus disease 2019 in Rio Grande, demonstrating that the COVID-19 pandemic exacerbated pre-existing social inequalities and exposed weaknesses in actions and public policies that should ensure food and nutritional security. The findings reinforced the urgency of public policies that adopt an intersectoral approach as a central axis for addressing food insecurity and strengthening social protection networks to prevent similar crises from further worsening the social insecurity scenario

## Data Availability

The anonymized database may be made available by the corresponding author upon request via email.
