# Supplementary material for: Incidence of food insecurity among adults and older adults diagnosed with coronavirus disease 2019: a longitudinal study, Rio Grande, 2021-2023
Source: Epidemiol Serv Saude. 2026 Aug 2;35:e20250935. doi: 10.1590/S2237-96222026v35e20250935.en (PMC13686312; doi:10.1590/S2237-96222026v35e20250935.en)
Supplement: Supplementary file 1 [file 2237-9622-ress-35-e20250935-Tab-sup-en.pdf]

Supplementary Table 1. Comparison of sociodemographic characteristics of participants according to follow-up status in the SulCovid-19 study. Rio Grande, 2021–2023 (n=2,897) Rio Grande, 2021–2023 (n=2,897)

Variable	Not followed up (n=970)	Followed up (n=1,927)	
	n (%)	n (%)	p-value
**Sex**			<0.001
Male	451 (37.7)	746 (62.3)	
Female	519 (30.6)	1180 (69.4)	
**Age group (years)**			<0.001
18–34	380 (40.0)	569 (60.0)	
35–59	441 (30.4)	1,012 (69.6)	
≥60	140 (29.2)	339 (70.8)	
**Race/color**			0.628
White	736 (33.1)	1487 (66.9)	
Brown (Brazilian mixed race)	121 (35.5)	220 (64.5)	
Black	94 (32.2)	198 (67.8)	
**Marital status**			0.469
Married/living with a partner	574 (32.9)	1168 (67.1)	
Single	271 (35.5)	493 (64.5)	
Separated	71 (30.5)	162 (69.5)	
Widowed	47 (33.8)	92 (66.2)	
**Educational level (years of schooling)**			0.459
0–8	236 (32.6)	487 (67.4)	
9–11	433 (34.5)	821 (65.5)	
≥12	277 (32.1)	586 (67.9)	
**Family income (BRL)**			0.254
Up to 1,000	220 (33.1)	445 (66.9)	
1,000-2,000	294 (29.6)	698 (70.4)	
2,000-4,000	205 (34.1)	397 (65.9)	
>4,000	91 (32.3)	191 (67.7)	
**Income during pandemic**			0.483
Stability	511 (33.4)	1,020 (66.6)	
Slight reduction	162 (30.9)	366 (69.1)	
Marked reduction	255 (34.0)	495 (66.0)	
**Employment status during pandemic**			0.539
Employment maintained	585 (33.9)	1143 (66.2)	
Pre-pandemic labor inactivity	273 (31.7)	589 (68.3)	
Unemployment due to the pandemic	58 (33.1)	117 (66.9)	
