# Supplementary material for: Incidence of food insecurity among adults and older adults diagnosed with coronavirus disease 2019: a longitudinal study, Rio Grande, 2021-2023
Source: Epidemiol Serv Saude. 2026 Aug 2;35:e20250935. doi: 10.1590/S2237-96222026v35e20250935.en (PMC13686312; doi:10.1590/S2237-96222026v35e20250935.en)
Supplement: Supplementary file 2 [file 2237-9622-ress-35-e20250935-Tab-sup-pt.pdf]

Tabela suplementar 1. Comparação das características sociodemográficas dos participantes segundo status de acompanhamento no estudo SulCovid-19. Rio Grande, 2021 e 2023 (n=2.897)

**Variável**	**Não acompanhados** (n=970)	**Acompanhados** (n=1.927)	
	**n (%)**	**n (%)**	**p-valor**
**Sexo**			<0,001
Masculino	451 (37,7)	746 (62,3)	
Feminino	519 (30,6)	1180 (69,4)	
**Faixa etária (anos)**			<0,001
18-34	380 (40,0)	569 (60,0)	
35-59	441(30,4)	1012 (69,6)	
≥60	140 (29,2)	339 (70,8)	
**Raça/cor da pele**			0,628
Branca	736 (33,1)	1487 (66,9)	
Parda	121 (35,5)	220 (64,5)	
Preta	94 (32,2)	198 (67,8)	
**Estado civil**			0,469
Casado(a)/Com companheiro(a)	574 (32,9)	1168 (67,1)	
Solteiro(a)	271 (35,5)	493 (64,5)	
Separado(a)	71 (30,5)	162 (69,5)	
Viúvo(a)	47 (33,8)	92 (66,2)	
**Escolaridade (anos)**			0,459
0-8	236 (32,6)	487 (67,4)	
9-11	433 (34,5)	821 (65,5)	
≥12	277 (32,1)	586 (67,9)	
**Renda familiar (reais)**			0,254
Até 1.000	220 (33,1)	445 (66,9)	
1.000-2000	294 (29,6)	698 (70,4)	
2.000-4.000	205 (34,1)	397 (65,9)	
>4.000	91 (32,3)	191 (67,7)	
**Renda durante pandemia**			0,483
Estabilidade	511 (33,4)	1020 (66,6)	
Redução leve	162 (30,9)	366 (69,1)	
Redução acentuada	255 (34,0)	495 (66,0)	
**Status de trabalho durante pandemia**			0,539
Emprego mantido	585 (33,9)	1143 (66,2)	
Inatividade laboral prévia	273 (31,7)	589 (68,3)	
Desemprego decorrente da pandemia	58 (33,1)	117 (66,9)	
