# Supplementary material for: Incidence of food insecurity among adults and older adults diagnosed with coronavirus disease 2019: a longitudinal study, Rio Grande, 2021-2023
Source: Epidemiol Serv Saude. 2026 Aug 2;35:e20250935. doi: 10.1590/S2237-96222026v35e20250935.en (PMC13686312; doi:10.1590/S2237-96222026v35e20250935.en)
Supplement: Translation

# Translation: Incidência de insegurança alimentar entre adultos e idosos diagnosticados com o coronavírus de 2019: estudo longitudinal, Rio Grande, 2021-2023

**DOI:** 10.1590/S2237-96222026v35e20250935.pt

**Authors:** Thais dos Anjos Velho, Lilia Schug de Moraes, Bianca Languer Vargas, Mirelle de Oliveira Saes, Suele Manjourany Silva Duro, Romina Buffarini

**Affiliations:** 1 Universidade Federal do Rio Grande, de Pós-Graduação em Ciências da Saúde, Rio Grande, RS, Brasil Universidade Federal do Rio Grande de Pós-Graduação em Ciências da Saúde Rio Grande RS Brasil; 2 Universidade Federal de Pelotas, Programa de Pós-Graduação em Enfermagem, Pelotas, RS, Brasil Universidade Federal de Pelotas Programa de Pós-Graduação em Enfermagem Pelotas RS Brasil

**Keywords:** Insegurança Alimentar, Covid-19, Adulto, Idoso, Estudos Longitudinais

## Abstract

**Objetivo:**

Avaliar a incidência de insegurança alimentar em um período de um ano e meio em Rio Grande, Rio Grande do Sul, e investigar fatores de risco, com a consideração de adultos e de idosos que tiveram a doença de coronavírus de 2019.

**Métodos:**

Trata-se de um estudo longitudinal, com coleta de dados em dois momentos: 2021 e 2022-2023. A insegurança alimentar foi avaliada por meio da versão reduzida da Escala Brasileira de Insegurança Alimentar. Foram consideradas como variáveis de exposição: sexo, idade, raça/cor, estado civil, grau de escolaridade, renda familiar, situação de renda e status de trabalho durante a pandemia. As variáveis foram descritas através de frequências absolutas e relativas, e as proporções foram comparadas com utilização do teste Qui-Quadrado. Os fatores associados foram estimados por regressão de Poisson com ajuste por variância robusta.

**Resultados:**

Entre os 1.231 indivíduos analisados, a incidência de insegurança alimentar no período foi de 20,1%. Na análise ajustada, verificou-se que o sexo feminino (razão de incidência [RI] 1,53; intervalo de confiança de 95% [IC95%] 1,21; 1,95). menores níveis de escolaridade (RI 1,75; IC95% 1,26; 2,41 e RI 1,54; IC95% 1,16; 2,04). menores níveis de renda familiar (RI 2,01; IC95% 1,17; 3,47 e RI 2,53; IC95% 1,54; 4,15) e redução acentuada da renda durante a pandemia (RI 1,51; IC95% 1,15; 1,98) foram fatores de risco associados à incidência de insegurança alimentar.

**Conclusão:**

Os resultados mostraram que dois em cada dez indivíduos desenvolveram insegurança alimentar ao longo do período de acompanhamento, que abrangeu a pandemia e o período subsequente, com maior risco entre indivíduos pertencentes a grupos socialmente mais vulneráveis.

**Aspectos éticos**

Esta pesquisa respeitou os princípios éticos, obtendo os seguintes dados de aprovação:

Comitê de ética em pesquisa: Universidade Federal do Rio Grande

Número do parecer: 4.375.697

Data de aprovação: 3/11/2020

Certificado de apresentação de apreciação ética: 39081120.0.0000.5324

Registro do consentimento livre e esclarecido: Obtido de todos os participantes antes da coleta.

## Introdução

No final de 2019, foi identificado na China um novo vírus respiratório, o coronavírus da síndrome respiratória aguda grave tipo 2, causador da doença do coronavírus de 2019^1^. Após a identificação dos primeiros casos e a confirmação do caráter contagioso da doença, o vírus se espalhou rapidamente pelo mundo, e a Organização Mundial da Saúde decretou, em março de 2020, a situação de pandemia. O Brasil, por sua vez, acatou as recomendações e passou a seguir os protocolos internacionais. Esse cenário desencadeou uma série de crises (sanitária, econômica, política e social). que contribuíram para o aumento da fome em todo o mundo^2^.

A insegurança alimentar e nutricional ocorre quando o indivíduo não tem acesso contínuo e permanente a uma alimentação saudável, de qualidade e em quantidades suficientes, que respeite a diversidade, a cultura local e aspectos sustentáveis. A Lei Orgânica de Segurança Alimentar e Nutricional (Lei nº 11.346, de 15 de setembro de 2006) estabelece que a segurança alimentar e nutricional é um direito básico que deveria ser garantido a todos os cidadãos, sem comprometer o acesso a outras necessidades essenciais^3^. Dado que a pandemia reduziu a produção e a distribuição de alimentos, isso impactou na oferta e na demanda, o que levou à redução do poder de compra da população, especialmente das famílias mais vulneráveis^4^.

Nesse cenário, tornou-se evidente a relação entre insegurança alimentar e renda familiar, com as famílias de baixa renda sendo as que mais enfrentam dificuldades para adquirir alimentos de qualidade e em quantidades diárias suficientes^5^. A redução na renda familiar decorrente da pandemia conduziu para uma mudança também nos hábitos alimentares da população adulta, como o aumento do consumo de alimentos ultraprocessados e, em alguns casos, a falta de alimento devido à extrema pobreza, caracterizada por renda insuficiente (renda per capita inferior a R$ 105,00)^6^.

Em 2018, a Pesquisa de Orçamentos Familiares evidenciou que aproximadamente 37% dos domicílios brasileiros já se encontravam em situação de insegurança alimentar^7^. O inquérito utilizou a Escala Brasileira de Insegurança Alimentar (EBIA) como medida direta da percepção da insegurança alimentar em nível domiciliar, categorizada em três níveis (leve, moderado e grave). A pesquisa demonstrou que a insegurança alimentar afetou a vida de milhões de brasileiros, o que levou ao destaque da importância de medidas e de políticas eficazes, bem como do apoio social para reduzir os impactos da fome e garantir a segurança alimentar e nutricional da população^7^.

De acordo com dados do Inquérito Nacional sobre Insegurança Alimentar no Contexto da Pandemia da doença do coronavírus de 2019 no Brasil, realizado pela Rede Brasileira de Pesquisa em Soberania e Segurança Alimentar e Nutricional, 55% dos domicílios brasileiros conviviam com algum grau de insegurança alimentar, e 19 milhões de pessoas passavam fome (nível grave) no período final de 2020^8^. Em pesquisa realizada em Rio Grande, Rio Grande do Sul, entre agosto de 2020 e fevereiro de 2021, foi identificado que a insegurança alimentar atingiu cerca de um terço dos domicílios^9^.

Este estudo buscou aprofundar a investigação sobre a insegurança alimentar no contexto da pandemia da covid-19 a partir de dois momentos em estudo longitudinal. No contexto brasileiro, não identificamos estudos que avaliaram a incidência de insegurança alimentar; a literatura nacional tem se concentrado predominantemente em estimativas de prevalência e em populações específicas^10^^-^^13^. Os objetivos deste trabalho foram, então, avaliar a incidência de insegurança alimentar em um período de um ano e meio que abrangeu a pandemia e um período subsequente (entre final de 2022 e início de 2023). em Rio Grande; e analisar os possíveis fatores de risco associados a adultos e idosos diagnosticados com a doença do coronavírus de 2019.

## Métodos

Delineamento do estudo

Trata-se de estudo longitudinal que utilizou dados da pesquisa SulCovid-19, inquérito de base populacional voltado ao monitoramento da saúde de adultos e de idosos após a infecção pela doença do coronavírus de 2019, conduzido em Rio Grande, no Rio Grande do Sul.

Contexto

Rio Grande fica localizada no extremo sul do Brasil e possuía, em 2022, uma população aproximada de 192 mil habitantes, com densidade demográfica de 71.53 habitantes/km² e índice de desenvolvimento humano de 0,74.

Os dados do estudo SulCovid-19 foram obtidos a partir de entrevistas estruturadas realizadas com adultos e idosos infectados pela doença do coronavírus de 2019 em Rio Grande. As coletas de dados ocorreram em dois momentos: (1) estudo de linha de base, de junho a outubro de 2021, que contou com amostra de 2.920 indivíduos, (2) estudo de acompanhamento, de outubro de 2022 a maio de 2023, com taxa de resposta de 66% (n=1.927). O intervalo médio entre as duas ondas do estudo foi de aproximadamente um ano e meio (17-18 meses), com a consideração dos pontos médios dos períodos de coleta.

A coleta foi realizada por entrevistadores previamente treinados. As respostas foram coletadas eletronicamente a partir do programa Research Eletronic Data Capture, com uso de tablets, e via chamadas telefônicas, com uso de smartphones. As chamadas foram gravadas com o auxílio de um aplicativo de celular gratuito (Callmaster), a fim de garantir a segurança do pesquisador e do entrevistado.

Participantes

A identificação da amostra foi realizada a partir da listagem de casos de doença de coronavírus de 2019 confirmados, obtida junto ao serviço de vigilância epidemiológica do município. A partir dessa listagem, os indivíduos que atendiam aos critérios de elegibilidade do estudo (n=3.822) foram contatados por telefone. Além das entrevistas por telefone, os entrevistadores também podiam optar por realizar a coleta de dados através de visitas domiciliares. No estudo, apenas um indivíduo foi entrevistado em domicílio.

Foram considerados elegíveis indivíduos com ≥18 anos, residentes em Rio Grande e diagnosticados com a doença do coronavírus de 2019 por meio do teste de transcrição reversa seguida de reação em cadeia de polimerase, entre dezembro de 2020 e março de 2021. Foram excluídos indivíduos com limitações funcionais e/ou doenças neurológicas avançadas que impedissem a resposta ao questionário, bem como aqueles que estavam em instituições de longa permanência ou privados de liberdade. Indivíduos que não foram localizados após cinco tentativas, uma por telefone, uma por WhatsApp e por três visitas domiciliares, foram considerados como perdas.

Variáveis

A variável de desfecho do estudo foi a incidência cumulativa de insegurança alimentar no domicílio. Para fins de simplificação textual, ela será referida ao longo do manuscrito apenas como incidência. As variáveis de exposição incluíram sexo, idade, raça/cor, estado civil, escolaridade, renda familiar, mudança de renda durante a pandemia e status de trabalho.

Medidas

A insegurança alimentar no domicílio foi coletada por meio da versão reduzida da EBIA, composta por cinco questões. O instrumento foi validado para a avaliação da insegurança alimentar na população brasileira e tem por objetivo identificar a percepção sobre a presença/falta de alimento no âmbito domiciliar. Neste estudo, as questões de insegurança alimentar foram realizadas para um único indivíduo no domicílio, que foi o entrevistado do SulCovid-19. A insegurança alimentar foi considerada presente quando o participante respondia "sim" a pelo menos um dos cinco itens do questionário.

A versão reduzida com cinco questões foi comparada à EBIA original de 14 questões (padrão-ouro) em uma amostra em Pelotas, Rio Grande do Sul, vizinho de Rio Grande^14^. O estudou mostrou alta sensibilidade (95,7%), especificidade de 100% e acurácia excelente (97%), com resultados semelhantes àqueles observados com a versão original.

Para o cálculo de incidência cumulativa de insegurança alimentar, em primeiro lugar, foi verificado o número de participantes com dados de insegurança alimentar nos estudos de linha de base e de acompanhamento (n=1.899). Foram excluídos do denominador indivíduos que já apresentavam o desfecho no estudo linha de base (n=668), pois não eram elegíveis para se tornarem casos novos. O denominador foram os indivíduos livres de desfecho no início do período (n=1.231). Foram, então, considerados casos incidentes aqueles indivíduos que não estavam em situação de insegurança alimentar no estudo linha de base e que passaram a relatar insegurança alimentar no acompanhamento. A incidência cumulativa correspondeu à proporção desses indivíduos que passaram a ter o desfecho ao longo do período entre a linha de base e o acompanhamento.

As variáveis de exposição foram coletadas no estudo de linha de base. Para fins de análise estatística, essas variáveis foram categorizadas da seguinte forma: sexo (masculino, feminino), faixa etária (18-34 anos, 35-59 anos, ≥60 anos), raça/cor (branca, parda, preta), estado civil (casado/com companheiro, solteiro, separado, viúvo), escolaridade (0-8 anos de estudo, 9-11 anos, ≥12 anos), renda familiar (menos de R$ 1.000,00, R$ 1.001,00 a R$ 2.000,00, R$ 2.001,00 a R$ 4.000,00, acima de R$ 4.000,00), situação da renda familiar durante a pandemia (estabilidade, redução leve, redução acentuada) e status de trabalho (emprego mantido, inatividade laboral prévia à pandemia e desemprego decorrente da pandemia).

A variável raça/cor foi coletada de acordo com as cinco categorias do Instituto Brasileiro de Geografia e Estatística: branca, preta, amarela, parda e indígena. Entre os indivíduos com dados no estudo de linha de base e no acompanhamento, o percentual de indivíduos autodeclarados amarelo foi de 0,3%, e indígena de 0,2%. Nenhuma observação dessas categorias foi elegível na amostra analítica final.

Tamanho da amostra

A amostra original compreendeu 2.920 indivíduos, dos quais 1.927 foram entrevistados no acompanhamento. Já que o estudo utilizou informações das duas etapas, a amostra analítica foi composta por 1.231 indivíduos com dados completos de insegurança alimentar no estudo de linha de base e de acompanhamento e que não estavam em risco de ficarem em situação de insegurança alimentar (Figura 1).

Viés

Estratégias de controle de viés foram adotadas antes e durante a coleta de dados. Essas estratégias incluíram a seleção criteriosa dos instrumentos de coleta, a elaboração de manuais operacionais, a padronização da equipe de entrevistadoras, a realização de estudo-piloto, além de controle rigoroso no gerenciamento e no processamento dos dados. Para reduzir perdas de acompanhamento, o estudo adotou uma estratégia ativa em duas etapas. Na coleta telefônica, cada participante elegível recebeu até cinco tentativas de contato, realizadas em dias e horários alternados, seguidas do recebimento de mensagem padronizada via WhatsApp, quando não havia resposta. Caso o indivíduo respondesse, a entrevista era agendada por telefone ou presencialmente, conforme sua preferência. Os participantes que não atendiam às chamadas nem respondiam às mensagens foram encaminhados para a etapa seguinte. Na coleta domiciliar, um entrevistador treinado e devidamente paramentado realizou ao menos uma visita presencial aos domicílios daqueles não entrevistados na fase telefônica. Endereços não localizados foram classificados como perdas.

**Figura 1.** Esquema de seleção da amostra de participantes a partir dos dados do estudo SulCovid-19. Rio Grande, 2021 e 2023

No momento da análise, para avaliar possível viés de seleção decorrente das perdas de acompanhamento, realizamos a comparação dos participantes acompanhados versus não acompanhados entre todos os elegíveis do estudo de linha de base (Tabela suplementar 1). A amostra de referência para essa comparação compreendeu 2.897 participantes, número obtido após a exclusão de 23 óbitos do estudo de linha de base (n=2.920). Na comparação entre participantes acompanhados e não acompanhados, observou-se maior proporção de mulheres e de indivíduos em faixas etárias mais elevadas (≥35 anos) entre os participantes acompanhados, enquanto as demais características sociodemográficas não diferiram entre os grupos (Tabela suplementar 1).

Métodos estatísticos

As variáveis foram descritas através de frequências absolutas e relativas, e, para variáveis ordinais, as proporções foram comparadas com a utilização do teste Qui-quadrado de Pearson ou teste do Qui-quadrado para tendência linear (Cochran-Armitage). A avaliação de possíveis fatores de risco associados à incidência de insegurança alimentar foi realizada com a utilização de modelos bruto e ajustado de regressão de Poisson com ajuste por variância robusta e estimação de razão de incidência (RI) e de seu respectivo intervalo de confiança de 95% (IC95%). Para evitar o ajuste por um possível mediador, as variáveis independentes foram incluídas no modelo ajustado em quatro níveis, de acordo com o modelo hierárquico de determinação^15^.

As variáveis independentes foram organizadas de acordo com suas distâncias conceituais ao desfecho e à possibilidade de influência causal entre os níveis. No primeiro nível, foram incluídas variáveis demográficas (sexo, idade, raça/cor); no segundo nível, foram incluídos estado civil, escolaridade e renda familiar, determinantes socioeconômicos que podem ser parcialmente explicados pelos níveis anteriores; no terceiro nível, foi incluída a variável status de trabalho; por último, foi incluída a de mudança de renda durante a pandemia, fator que pode ter sido condicionado pelos níveis anteriores e pode estar diretamente relacionado às condições recentes de acesso a alimentos. Associações com p-valor<0,050 foram consideradas estatisticamente significantes. Todas as análises foram realizadas com a utilização do software Stata 16.1 (StataCorp).

## Resultados

A Tabela 1 apresenta as características sociodemográficas da amostra analítica (n=1.231). A maioria dos participantes era do sexo feminino (56,2%), quase metade era da faixa etária 35-59 anos (52,3%), a maioria se autodeclararam brancos (80,7%) e eram casados ou com companheiro(a) (64,2%). Em relação à escolaridade, a maior proporção apresentou entre 9-11 anos de estudo (42,9%), e 57,3% relataram renda familiar mensal de até R$ 2.000. Durante a pandemia, quase um terço (34,4%) referiram algum nível de redução de renda, enquanto 4,1% relataram ter ficado desempregados em decorrência da pandemia. Divergências no total das variáveis em relação ao número total de observações na amostra analítica podem ter sido devido a informações perdidas de algumas variáveis: renda familiar (10,7%), raça/cor da pele (4,7%), status de trabalho durante a pandemia (3,6%), escolaridade (1,6%), renda durante pandemia (2,4%), estado civil (0,4%) e idade (0,1%).

**Tabela 1:** Descrição da amostra analítica com dados provenientes do estudo SulCovid-19. Rio Grande, 2021 a 2023 (n=1.231)

Variável	n (%)
Sexo	
Masculino	539 (43,8)
Feminino	692 (56,2)
Faixa etária (anos)	
18-34	350 (28,5)
35-59	643 (52,3)
≥ 60	236 (19,2)
Raça/cor	
Branca	938 (80,7)
Parda	132 (10,8)
Preta	103 (8,5)
Estado civil	
Casado(a)/com companheiro(a)	787 (64,2)
Solteiro(a)	285 (23,2)
Separado(a)	95 (7,8)
Viúvo(a)	59 (4,8)
Escolaridade (anos)	
0-8	263 (21,8)
9-11	520 (42,9)
≥12	428 (35,3)
Renda familiar (reais)	
Até 1,000	205 (18,7)
1,001-2,000	424 (38,6)
2,001-4,000	301 (27,4)
≥4,001	169 (15,3)
Renda durante pandemia	
Estabilidade	792 (65,6)
Redução leve	218 (18,1)
Redução acentuada	196 (16,3)
Status de trabalho durante a pandemia	
Emprego mantido	79 (65,7)
Inatividade laboral prévia	358 (30,2)
Desemprego decorrente da pandemia	49 (4,1)

**Tabela 2:** Incidência de insegurança alimentar em uma amostra de adultos e idosos segundo variáveis sociodemográficas. SulCovid-19, Rio Grande, 2022 e 2023 (n=1.231)

Variável	Insegurança alimentar (%)	p-valor
Overall	20,1	
Sexo		<0,001
Masculino	15,6	
Feminino	23,6	
Faixa etária (anos)		0,789
18-34	18,9	
35-59	20,7	
≥60	19,9	
Raça/cor		0,258
Branca	19,5	
Parda	18,9	
Preta	26,2	
Estado civil		0,194
Casado(a)/com companheiro(a)	18,2	
Solteiro(a)	22,8	
Separado(a)	25,3	
Viúvo(a)	20,3	
Escolaridade (anos)		0,003
0-8	23,6	
9-11	21,9	
≥12	15,0	
Renda familiar (reais)		<0,001
Até 1,000	22,4	
1,001-2,000	26,2	
2,001-4,000	16,3	
≥4,001	9,5	
Renda durante pandemia		0,001
Estabilidade	17,6	
Redução leve	22,9	
Redução acentuada	27,0	
Status de trabalho durante a pandemia		0,283
Emprego mantido	19,1	
Inatividade laboral prévia	22,1	
Desemprego decorrente da pandemia	26,5	

**Table 3:** Risco relativo (RR) e intervalos de confiança de 95% (IC95%) brutos e ajustados da incidência de insegurança alimentar pelas variáveis sociodemográficas do estudo. Sul Covid-19, Rio Grande, 2021 e 2023 (n=1.899)

Variável	Bruta		Ajustada	
	RR (IC95%)	p-valor	RR (IC95%)	p-valor
Sexo		<0,001		<0,001
Masculino	Ref.			
Feminino	1,51 (1,19; 1,92)		1,53 (1,21; 1,95)	
Faixa etária (anos)		0,79		0,761
18-34	0,95 (0,68; 1,32)		0,92 (0,66; 1,31)	
35-59	1,03 (0,77; 1,39)		1,02 (0,76; 1,39)	
≥60	Ref.			
Raça/cor		0,237		0,192
Branca	Ref.		Ref.	
Parda	0,97 (0,67; 1,41)		0,99 (0,67; 1,45)	
Preta	1,34 (0,95; 1,90)		1,38 (0,97; 1,95)	
Estado civil		0,187		0,107
Casado(a)	Ref.		Ref.	
Solteiro(a)	1,26 (0,97; 1,62)		1,30 (1,00; 1,69)	
Separado(a)	1,39 (0,95; 2,02)		1,34 (0,91; 1,94)	
Viúvo(a)	1,11 (0,66; 1,89)		0,84 (0,47; 1,49)	
Escolaridade (anos)		0,007		0,001
0-8	1,58 (1,15; 2,16)		1,75 (1,26; 2,41)	
9-11	1,47 (1,11; 1,94)		1,54 (1,16; 2,04)	
≥12	Ref.		Ref.	
Renda familiar (reais)		<0,001		<0,001
Até 1,000	2,37 (1,39; 4,03)		2,01 (1,17; 3,47)	
1,001-2,000	2,77 (1,69; 4,53)		2,53 (1,54; 4,15)	
2,001-4,000	1,72 (1,01; 2,92)		1,66 (0,97; 2,83)	
≥4,001	Ref.		Ref.	
Renda na pandemia		0,005		0,009
Estabilidade	Ref.		Ref.	
Redução leve	1,31 (0,98; 1,74)		1,28 (0,95; 1,99)	
Redução acentuada	1,54 (1,17; 2,02)		1,51 (1,15; 1,98)	
Status de trabalho durante a pandemia		0,271		0,715
Emprego mantido	Ref.		Ref.	
Inatividade laboral prévia	1,15 (0,91; 1,47)		0,90 (0,66; 1,21)	
Desemprego decorrente da pandemia	1,39 (0,85; 2,25)		1,05 (0,62; 1,81)	

A incidência de insegurança alimentar na amostra total foi de 20,1% (Tabela 2). Quando analisada por sexo, foi significativamente maior entre as mulheres (23,6%) em comparação aos homens (p-valor<0,001) e entre pessoas com 0-8 anos de escolaridade (23,6%) em relação a participantes com maior nível de escolaridade (15,0%) (p-valor 0,006). Não houve diferenças significativas segundo as faixas etárias (p-valor 0,789). a raça/cor (p-valor 0,258) ou o estado civil (p-valor 0,194). Observou-se maior incidência de insegurança alimentar em indivíduos com renda familiar mensal de até R$ 2.000 (p-valor<0,001). A incidência apresentou uma redução progressiva à medida que os níveis de escolaridade aumentavam (p-valor de tendência linear 0,003). Foi observado um gradiente para mudança de renda na pandemia, com registro de maior incidência de insegurança alimentar entre aqueles que declararam ter redução acentuada, seguida por redução leve, em relação a quem manteve sua renda estável (p-valor de tendência linear 0,001). Verificou-se também que a incidência de insegurança alimentar foi maior entre participantes que ficaram desempregados em decorrência da pandemia (26,5%), seguido pelos inativos laboralmente (22,1%), porém, esta associação não foi significativa (p-valor 0,283) (Tabela 2).

Nas análises brutas e ajustadas, as variáveis sexo, escolaridade, renda familiar e mudança de renda na pandemia estiveram associadas à incidência de insegurança alimentar (Tabela 3). Os resultados mostraram que mulheres (RR 1,53; IC95% 1,21; 1,95); aqueles com menos anos de escolaridade (0-8 anos: RR 1,75; IC95% 1,26; 2,41; 9-11 anos: RR 1,54; IC95% 1,16; 2,04); com menor renda familiar (até R$1.000: RR 2,01; IC95% 1,17; 3,47; entre R$ 1.001-2.000: RR 2,53; IC95% 1,54; 4,15) e aqueles que relataram redução acentuada de renda durante a pandemia (RR 1,51; IC95% 1,15; 1,98) apresentaram maior risco de insegurança alimentar (Tabela 3).

## Discussão

Os dados do presente estudo revelaram que entre adultos e idosos diagnosticados com a doença do coronavírus de 2019 em Rio Grande, dois em cada dez desenvolveram insegurança alimentar ao longo de período de aproximadamente um ano e meio (entre 2021 e final de 2022/início de 2023), mesmo após o período mais crítico da pandemia de covid-19. Mulheres, aqueles com menor escolaridade, com menor renda familiar e aqueles que relataram redução da renda durante a pandemia, apresentaram maior risco de incidência de insegurança alimentar. 

A escassez de estudos longitudinais que avaliaram a incidência de insegurança alimentar durante a pandemia limitou algumas comparações diretas com as análises deste estudo. Em pesquisa realizada no sul do estado, por meio de quatro inquéritos epidemiológicos sobre a doença do coronavírus de 2019, foi verificado que cerca de um terço dos domicílios enfrentavam situações de insegurança alimentar em 2020^16^. Embora tenham sido dados transversais, essas evidências corroboraram os achados do presente estudo e contextualizaram a magnitude do problema na região.

O Inquérito Nacional da Rede Brasileira de Pesquisa em Soberania e Segurança Alimentar e Nutricional mostrou que mais da metade dos domicílios brasileiros (55,2%) vivia algum grau de insegurança alimentar no contexto pandêmico, mas sem dados de incidência^17^. Em todas as regiões do estado, a desigualdade de renda foi o fator que mais contribuiu para as condições de segurança alimentar e nutricional, e as famílias com menor renda foram as mais vulneráveis à insegurança alimentar, como evidenciado também no presente estudo^8^. Em estudo realizado em Porto Alegre, foi identificado que 33% das pessoas entrevistadas estavam em situação de insegurança alimentar e nutricional, e os grupos mais atingidos incluíam mulheres chefes de família, pessoas com baixa escolaridade e famílias com renda menor que três salários mínimos^18^.

A literatura tem evidenciado que os impactos sociais e econômicos provocados pela pandemia contribuíram para o aumento de insegurança alimentar nos domicílios^19^. Esse contexto também destacou desigualdades estruturais históricas do Brasil^20^^,^^21^. com reflexos do racismo estrutural, da organização social patriarcal e do enfraquecimento de políticas públicas que limitam o acesso a oportunidades de trabalho, de renda e de educação. A redução na renda, o aumento do desemprego e a ausência de políticas e de programas de assistência acentuaram o contexto de desigualdade social preexistente, bem como o cenário de insegurança alimentar e nutricional^7^^,^^17^. O desmonte das políticas públicas de segurança alimentar e nutricional no Brasil^22^ contribuiu para o agravamento alimentar dessa conjuntura. A extinção do Conselho Nacional de Segurança Alimentar e Nutricional, a desarticulação da Câmara Interministerial de Segurança Alimentar e Nutricional e a redução de programas voltados à área comprometeram o financiamento e a execução de ações essenciais, o que enfraqueceu a soberania alimentar e aumentou a vulnerabilidade social^4^.

As descobertas deste estudo possuem implicações práticas relevantes, sobretudo por se tratar do contexto pandêmico da covid-19, cujos impactos sociais, embora já conhecidos e debatidos, ainda geram repercussões sociais que persistem atualmente na sociedade e contribuem para a pauta de direitos sociais. Esses resultados fornecem subsídios importantes para estratégias locais e nacionais que visem o aprimoramento de políticas públicas voltadas para a segurança alimentar e nutricional, a saúde e a assistência social, especialmente entre os grupos mais vulneráveis. Pesquisas indicaram que a recuperação econômica precisa ser acompanhada de medidas intersetoriais que garantam o acesso universal e equitativo à alimentação e à garantia de direitos sociais^4^^,^^23^.

O estudo apresentou limitações, especialmente relacionadas às perdas de acompanhamento. A maior proporção de mulheres e de indivíduos de faixas de idade mais elevadas entre os acompanhados pode ter influenciado as estimativas, já que esses grupos são mais vulneráveis à insegurança alimentar^5^^,^^24^. O fato também de a amostra ter sido composta apenas por indivíduos infectados com a doença do coronavírus de 2019 restringiu a generalização desses achados para populações quem não sofreram o impacto da infecção pelo vírus da covid-19. A mesma limitação se aplica ao fato de o estudo ter sido conduzido em Rio Grande, o que limita a validade externa dos resultados para outras regiões com contextos socioeconômicos e culturais diferentes. Os resultados, assim, devem ser interpretados com cautela. A subnotificação de casos de covid-19 no Brasil^25^ pode ter levado à identificação de número menor de indivíduos infectados na amostra original do estudo. Isso pode limitar a representatividade da amostra em relação ao total de pessoas infectadas na população.

Ressalta-se, porém, que isso não compromete as estimativas de insegurança alimentar entre os participantes incluídos, uma vez que o estudo se baseou exclusivamente em casos confirmados por RT-PCR e amplamente captados pela vigilância municipal. Acredita-se que os procedimentos rigorosos de tentativa de contato, com inclusão de múltiplas ligações telefônicas, do envio de mensagens via WhatsApp e de visita domiciliar, minimizaram substancialmente a subnotificação dentro do estudo. Por último, é importante salientar que, além das variáveis analisadas neste estudo, outros fatores como rede de apoio social^26^^,^^27^, recebimento de auxílio emergencial^28^^,^^29^ e arranjos domiciliares^24^ têm sido associados à insegurança alimentar e devem ser explorados em estudos futuros. Esses aspectos podem impactar diretamente a capacidade das famílias brasileiras de enfrentarem situações de vulnerabilidade.

Entre os pontos fortes, destaca-se o delineamento longitudinal, com o qual conseguimos mostrar a incidência de insegurança alimentar ao longo do tempo e a possibilidade de registro da insegurança alimentar regional, mesmo com a limitação da amostra, conduzida no período pandêmico e início do pós-pandêmico, possibilitando um levantamento da situação em Rio Grande durante um momento importante de crise sanitária.

Os dados deste estudo mostraram a incidência de insegurança alimentar entre adultos e idosos diagnosticados com a doença do coronavírus de 2019 em Rio Grande, o que ajudou a evidenciar que a pandemia de covid-19 foi fator agravante de desigualdades sociais preexistentes e que expôs fragilidades nas ações e nas políticas públicas que deveriam garantir a segurança alimentar e nutricional. Os dados reforçaram a urgência de políticas públicas que considerem a intersetorialidade como eixo principal para o enfrentamento da insegurança alimentar e que fortaleçam redes de proteção social para evitar que crises semelhantes agravem ainda mais o cenário de insegurança social.

## Data Availability

O banco de dados anonimizado pode ser disponibilizado pelo autor correspondente mediante solicitação por e-mail.

## Material Suplementar

Tabela Suplementar
